# High-density sub-100-nm peptide-gold nanoparticle complexes improve vaccine presentation by dendritic cells *in vitro*

**DOI:** 10.1186/1556-276X-8-72

**Published:** 2013-02-12

**Authors:** Adam Yuh Lin, Jessica Lunsford, Adham Sean Bear, Joseph Keith Young, Phillip Eckels, Laureen Luo, Aaron Edward Foster, Rebekah Anna Drezek

**Affiliations:** 1 Department of Bioengineering, Rice University, 77005, Houston, TX USA; 2 Center for Cell and Gene Therapy, Baylor College of Medicine, 77030, Houston, TX, USA; 3 Department of Electrical and Computer Engineering, Rice University, 77005, Houston, TX USA

**Keywords:** Vaccines, Gold nanoparticles, ELISPOTs, Immunotherapy, Dendritic cells, Self-assembled monolayer

## Abstract

Nanocarriers have been explored to improve the delivery of tumor antigens to dendritic cells (DCs). Gold nanoparticles are attractive nanocarriers because they are inert, non-toxic, and can be readily endocytosed by DCs. Here, we designed novel gold-based nanovaccines (AuNVs) using a simple self-assembling bottom-up conjugation method to generate high-peptide density delivery and effective immune responses with limited toxicity. AuNVs were synthesized using a self-assembling conjugation method and optimized using DC-to-splenocyte interferon-γ enzyme-linked immunosorbent spot assays. The AuNV design has shown successful peptide conjugation with approximately 90% yield while remaining smaller than 80 nm in diameter. DCs uptake AuNVs with minimal toxicity and are able to process the vaccine peptides on the particles to stimulate cytotoxic T lymphocytes (CTLs). These high-peptide density AuNVs can stimulate CTLs better than free peptides and have great potential as carriers for various vaccine types.

## Background

Cancer immunotherapy is a promising new strategy that stimulates the patients' immune systems to target and kill tumors. Various groups have investigated enhancing the induction of anti-tumor T cell responses through vaccines, including immunization with tumor-associated antigens (TAA) or TAA-derived peptides [1-4]. In phase I trials, gp100 peptides, a melanocyte lineage-specific protein expressed in most melanomas, elicited strong anti-melanoma CD8^+^ cytotoxic lymphocytes (CTLs) (in 14% of patients) and CD4^+^ helper T cell effects (in 79% of patients) [5,6]. In phase III trials, gp100 vaccination showed favorable progression-free survival for metastatic melanoma [7]. However, the overall response rate was only 2.6% with this peptide vaccine method, and the responses were transient [2]. This low response could be attributed to peptide degradation and diffusion, limiting the amount of antigen delivered to the peripheral antigen-presenting cells (APCs), including lymph node-resident dendritic cells (DC).

To address these issues, several nanocarriers have been explored to improve the delivery of tumor antigens to DCs. The four main types of nanoparticles that have been explored in this capacity are liposomal, viral-based, polymer-based, and metallic particles [8]. Commonly used polymeric and liposomal nanoparticles have two main limiting factors. First, liposomal and polymeric particles can be toxic under high doses due to membrane fusion and acidic monomers, respectively [8]. Second, these particles are greater than 100 nm in diameter and stay at the injection site, requiring peripheral DCs to migrate to the lymph nodes for exposure to the vaccine antigens [9], whereas smaller nanoparticles (approximately 45 nm) have been reported to drain into lymph nodes and are readily taken up by DCs following subcutaneous (s.c.) injections [9,10]. These studies indicate that sub-100-nm nanocarrier designs can facilitate antigen delivery to professional APCs in the lymph nodes.

Gold nanoparticles (AuNPs) are inert, non-toxic, and can be readily endocytosed by DCs and other phagocytic mononuclear cells [11-13]. *In vitro* studies have demonstrated that even non-phagocytic T cells can load up to 10^4^ particles per cell [14]. The capacity for AuNPs to be uptaken by cells may allow improved delivery of antigens and therefore improve the overall vaccine antigen dose delivered to APCs. Additionally, modifications of AuNPs are straightforward as molecules with free thiols can self-assemble into a monolayer on the gold surface by forming strong gold-sulfide dative bonds. This provides an efficient and cost-effective platform for antigen delivery. Although most vaccines use subcutaneous injections, gold nanoparticles tend to accumulate in the reticulo-endothelial system when injected intravenously (i.v.) [15]. For other AuNP-based drug delivery systems, this phenomenon is commonly viewed as potentially toxic or can result in adverse side effects. However, for vaccine delivery, particle accumulation in the spleen can be very advantageous because it is the largest immune organ in the body containing significant numbers of lymphocytes and APCs. Therefore, gold nanovaccines (AuNVs) can potentially improve the efficacy of both i.v. and s.c. vaccines.

**Figure 1:**
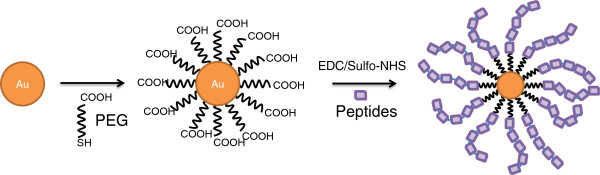
**Schematic of gold-based nanovaccine design synthesis.** The AuNPs were coated with self-assembled monolayers of 5000-MW PEG-SH. The AuNPs were subsequently conjugated with the desired peptides using EDC and sulfo-NHS as linkers.

Most liposomal and polymer formulations use encapsulation methods to incorporate vaccine peptides. Making smaller particles using this method reduces the peptide load delivered to innate immune cells. Conventionally, vaccine antigen AuNP complexes are assembled in two ways: (1) direct conjugation of the peptides onto the gold surface using the thiols on the cysteine residues or (2) electrostatic binding of the peptides onto modified or unmodified gold surfaces [8,16,17]. However, these methods only allow one layer of peptides or form aggregates electrostatically on the gold surfaces. Thus, we devised a method to self-assemble large quantities of peptides on AuNPs using a simple 1-ethyl-3-(3-dimethylaminopropyl) carbodiimide (EDC) and sulfo-*N*-hydroxysuccinimide (sulfo-NHS) bottom-up conjugation strategy to layer peptides onto modified AuNPs (Figure 1). The tandem repeats of peptides, incorporated onto this AuNV design, have shown improved vaccination efficacy in non-gold particle systems [18,19]. Furthermore, the simple bottom-up conjugation design can allow effective delivery of large doses of vaccine peptides and thus improve immunogenicity of the vaccine antigen peptides. Here, we evaluated the high-peptide density AuNVs through three steps: synthesis and characterization, AuNV uptake by dendritic cells, and functional *in vitro* immunologic assays.

## Methods

### Reagents

All of the polyethylene glycol (PEG) products were purchased from NanoCS (New York, NY, USA). The citrate-stabilized gold colloids were purchased from Ted Pella (Redding, CA, USA). All of the buffers and chemicals were purchased from Sigma-Aldrich (St. Louis, MO, USA), Thermo Scientific (Waltham, MA, USA), and Invitrogen (Carlsbad, CA, USA). The peptides were purchased from Genemed Synthesis (San Antonio, TX, USA). The JAWS II cells and media were purchased from ATCC (Manassas, VA, USA).

### Two-step AuNV synthesis

First, carboxyl-PEG-thiols were added to a 30-nm gold colloid solution (2 × 10^11^ particles/ml) with an end concentration of 5 μM and incubated for 24 h. The solution was raised to 0.1 M NaCl, 10 mM sodium phosphate, and 0.1% Tween 20. The excessive PEG molecules were removed from the AuNP solution by three centrifugation-washing steps at 7,000×*g* for 20 min with phosphate-buffered saline (PBS). The final particle pellet was diluted with 2-(*N*-morpholino)ethanesulfonic acid (MES) buffer. EDC (4.25 mg) and sulfo-NHS linker (6.4 mg) were added to the particle-MES solution and incubated for 15 min at room temperature. The excessive linkers were removed from the solution by centrifuging in a 10,000 molecular weight cutoff filter at 2,000×*g* for 15 min and diluting the particles with PBS. The peptides (50 μg) were then added to the particles per milliliter of solution, and the mixture was incubated for 30 min, 1 h, 2 h, and 24 h at room temperature. Varying the incubation time was for optimization of the conjugation scheme. Hydroxylamine (10 mM) was added to quench any unbound EDC/NHS for an additional hour. The peptide-coated particles were then centrifuged and washed three times with PBS. After the final PBS wash/centrifuge cycle, the supernatant was removed, and the particle pellet was re-suspended in 200 μl of PBS. The sample was sonicated and stored at 4°C until used.

### One-step AuNV synthesis

After the carboxyl-PEG-AuNPs were suspended in MES buffer, 5 μl of 44 mM EDC and 5 μl of 59 mM sulfo-NHS linkers were added per milliliter of particle-MES solution and incubated for 15 min at room temperature. The peptides (50 μg) were then added to the particles per milliliter of solution, and the mixture was incubated for 1 h. Hydroxylamine (10 mM) was added to quench any unbound EDC/NHS for an additional hour. The collection process was the same as before. To assess gold nanoparticle core size on AuNV efficacy, 15-nm and 80-nm AuNPs were used to synthesize AuNVs. For the 15-nm and 80-nm AuNVs, the stock particle concentration started at 1.4 × 10^12^ and 1.1 × 10^10^ particles/ml, respectively, as provided by Ted Pella. The conjugation process was the same.

### Splenocyte harvest protocol

C57BL/6J, pmel-1, and OT-I mice (Jackson Laboratories, Bar Harbor, ME, USA) were maintained in the pathogen-free mouse facility at Baylor College of Medicine. This study was approved by the Institutional Animal Care and Use Committees (IACUC) of Baylor College of Medicine (# A-3823-01). The spleens were harvested from pmel-1 mice and homogenizing the tissue through a cell strainer formed a single cell suspension. The cells were collected, and the red blood cells (RBCs) were lysed to yield a suspension of splenocytes (2 M/ml) and used within an hour of harvesting. The OT-I splenocytes were collected through the same method and were frozen until use in the enzyme-linked immunosorbent spot (ELISPOT) assays.

### Bone marrow-derived dendritic cell harvest and exposure protocol

The femur and tibia from both sides of a C57BL/6 mouse were harvested and flushed into a petri dish. After lysing the RBCs, the cells were grown on a 10-cm dish for 48 h at 37°C in bone marrow-derived dendritic cell (BMDC) media supplemented with IL-4 and GM-CSF. After 2 days, the media was aspirated, and fresh media was added to the dish for another 2 days. Then, BMDCs were collected by vigorously rinsing the dish and plated onto 12-well plates at 2 M cells per well. After 24 h, the AuNVs and other conditions were added to each well for another 24 h. The BMDCs were then washed with PBS to remove any free particles and diluted to 500,000 cells/ml.

### Interferon-γ ELISPOT

Splenocytes (200,000) were added to 96-well plates that were pre-coated with anti-interferon-γ (IFN-γ) antibodies. Free AuNVs or 50,000 loaded BMDCs were added to each well and incubated for 24 h at 37°C. The cells were decanted, and then the plate was washed with PBS/0.05% Tween 20 six times. Biotinylated anti-IFN-γ antibodies were added to the plate to form sandwich assays for 2 h at 37°C. After washing excess antibodies off the plate, avidin-peroxidase complexes (Vectastain, Vector Laboratories, Burlingame, CA, USA) were added to the plates to bind to the biotin molecules. Spots were developed by adding 3-amino-9-ethylcarbazole (AEC) and hydrogen peroxide. The dried membrane was punched out of the plate, and spots were evaluated by ZellNet Consulting (Fort Lee, NJ, USA).

### Conjugation yield calculation

Trp-2 (1 μg/μl) peptides were diluted into 14 different concentrations. The 280-nm absorbance values of the Trp-2 peptides were used to generate a concentration standard curve. The peak absorbance values in the visible range (400 to 800 nm) from the dilutions of the 30-nm gold colloid stock (2 × 10^11^ particles/ml) were used to plot against the 280-nm absorbance values. The actual 280-nm absorbance of the Trp-2 peptides was measured by calculating the difference between the Trp-2 peptide 280-nm absorbance values for the Trp-2 AuNVs and the standardized 280-nm values from the 30-nm gold colloids. The peptide concentration was calculated by correlating the absorbance values to the Trp-2 standard curve (Additional file 1: Figure S1).

### Toxicity test protocol

One-hundred microliters of JAWS II cells, a BMDC cell line, were added to a 96-well plate (500,000 cells/ml). Ovalbumin (OVA) or gp100 AuNVs (1 to 10 μl of 10^11^particles/ml) were added to the cells for 24 h at 37°C. Ten microliters of alamarBlue (Life Technologies Corporation, Carlsbad, CA, USA) was then added to each well and incubated for 2 h at 37°C. The fluorescent readings at 585 nm (excited at 570 nm) were measured with a Fluorolog-3 plate reader.

### Lysate degradation study

From the one-step AuNV protocol, 25 μg of fluorescein isothiocyanate (FITC) fluorescent peptides were added to the solution prior to hydroxylamine. This step allows the fluorescent peptides to be on the outside layer of the AuNVs. JAWS II cells (500,000) were lysed in 1 ml CHAPS lysis buffer. The particles (10^11^) were added to either the CHAPS lysis buffer or to the JAWS II lysate for 24 h. The particles were removed by centrifuging at 7,000×*g* for 20 min. The supernatants were transferred to a 96-well plate, and the FITC fluorescence was measured at 520 nm (excitation at 485 nm).

## Results and discussion

### Self-assembled AuNV particle synthesis

First, carboxyl-PEG-thiols were self-assembled onto citrate-stabilized 30-nm gold colloids to form a monolayer. PEG was chosen for its bio-inert and non-toxic properties and the ability to protect AuNPs during the conjugation process [20]. Next, EDC and sulfo-NHS linkers in MES buffer were added to the particle solution for carboxyl activation.

Following the suggested protocol adapted from Grabarek and Gergely [21], the majority of the excess linkers were then removed from the solution via a centrifuge filter. The particles were transferred to PBS buffer, and the vaccine peptides or hydroxylamine (control) were subsequently added. This two-step method is best known to allow coupling of the two proteins without strongly affecting the second protein's carboxyls. Three MHC class I peptides were used: one from model antigen OVA (SIINFEKL) and two from melanoma antigens, gp100 (KVPRNQDWL) and Trp-2 (SVYDFFVWL) [22,23].

**Figure 2:**
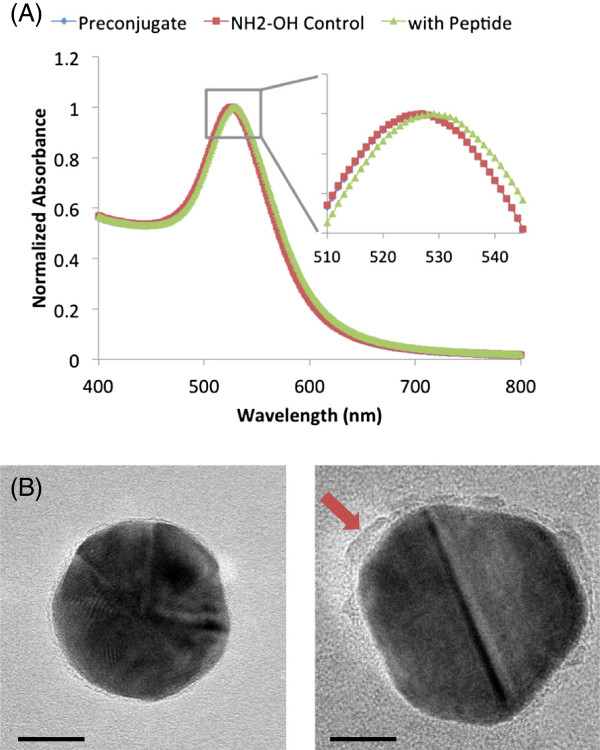
**Characterization of AuNV conjugation process.** (**A**) The absorbance spectra of the initial peptide AuNP conjugates. The full view shows the 400- to 800-nm range, and the zoom insert shows the peaks between 510 and 545 nm. Preconjugate refers to the carboxyl-PEG-AuNPs. The NH_2_OH control refers to capping the active carboxyl groups on the particles with hydroxylamine. The preconjugates and NH_2_OH control particles had the same peak, verifying that the conjugation protocol does not alter the absorbance peak. The particles conjugated with peptides show a 2-nm red shift. (**B**) TEM images of a 30-nm AuNP coated with PEG and a 30-nm gp100 AuNV. The surface of the peptide-coated AuNV appears rougher and thicker (red arrow) than the PEG-coated AuNP, indicating successful conjugation (scale bar = 10 nm).

Peptide conjugation was verified by measuring the optical extinction spectra for preconjugated particles (PEG-coated 30-nm gold colloids), hydroxylamine (NH_2_OH) particles, and gp100 (KVPRNQDWL) AuNVs. The NH_2_OH particles (negative controls) underwent the same synthesis procedures as the gp100 AuNV particles, but NH_2_OH was added instead of peptides to quench the EDC reaction. This control particle accounted for any spectral changes due to the entire conjugation process. The AuNP peak absorbance red shifted from 523 to 527 nm when carboxyl-PEG-SH bound to the particle surface. When the gp100 peptides were conjugated to the AuNPs, the peak shifted further to 529 nm, indicating successful peptide conjugation onto the nanoparticle surface. The hydroxylamine control particles' extinction peak did not red shift, indicating that the red shift of the AuNV absorbance spectra is not a result of the conjugation process alone, but is caused by the peptide linkage (Figure 2A).

In Figure 2B, the particles were dried prior to transmission electron microscopy (TEM) imaging, so the normally hydrated PEG molecules collapsed onto the AuNP surface, showing a uniform light rim around the border of the gold particle (Figure 2B). Post-peptide conjugation, the AuNV TEM images showed thickening and rough edges on the AuNP surface, which can be caused by peptide linkage to the PEG molecule and self-polymerization.

### AuNV characterization

Particle size is important for lymphatic drainage from the injection site, biodistribution, and cellular endocytosis. Dynamic light scattering measurements (DLS) showed that the OVA AuNVs were less than 80 nm in diameter, which is much smaller than other liposomal or polymeric formulations and, therefore, can potentially improve lymphatic drainage when injected subcutaneously. The zeta potentials correlate well with the free-peptide properties because the gold colloids and COOH-PEG-AuNPs were capped with either citrate or carboxyls; however, the OVA AuNVs show near-neutral potentials because the OVA peptides have no charge at physiologic pH (Table 1).

**Table 1:** DLS results, polydispersity index, and zeta potentials of citrate-capped gold colloids, COOH-PEG-coated AuNPs, and OVA AuNVs

	**Size (nm)**	**PDI**	**Zeta (mV)**
Colloids	33.5 ± 6.3	0.124	−37.6 ± 6.5
COOH-PEG-AuNPs	61.5 ± 6.2	0.201	−27.6 ± 12.2
OVA AuNVs	77.9 ± 9.5	0.305	−0.7 ± 6.7

For conjugation yield approximation, Trp-2 peptides were used due to the high absorbance of the Y amino acid at 280 nm. The Trp-2 AuNVs were calculated to have 24.6 μg of peptide per 10^11^ particles based on UV–vis absorbance measurements. After subtraction of the standard curves, the conjugation yield was calculated to be approximately 90% (Additional file 1: Figure S1).

### Dendritic cell uptake of AuNVs

**Figure 3:**
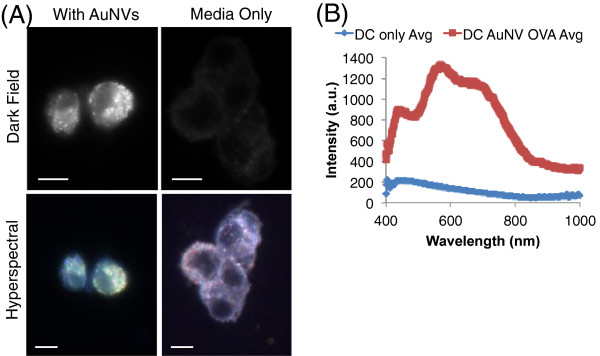
**Image and hyperspectral analysis of BMDC loaded AuNVs.** (**A**) Dark-field and hyperspectral images of DCs loaded with AuNVs or DCs only. Only DCs loaded with AuNVs appeared in the dark-field images with the same exposure time. The hyperspectral images show a spectral shift from purple blue to yellow green when the DCs were loaded with AuNVs (scale bars = 10 um). (**B**) The average spectral data for BMDCs with or without AuNVs, using each cell as regions of interest. The intensities were calibrated to the lamp spectra baseline.

After characterization of the AuNVs, the next step was to evaluate their interaction with dendritic cells. Using dark-field imaging, the DCs loaded with AuNVs showed significantly more scattering due to the AuNPs compared to untreated DCs with the same imaging exposure (4 ms). The hyperspectral data showed that the loaded DCs had a spectral shift toward 550 nm, close to the absorbance peak at 529 nm of AuNVs in solution, suggesting that the enhanced scattering was caused by AuNPs (Figure 3). The shift in the peak plasmon resonance wavelength of AuNVs in cells compared to that in solution may be attributed to the higher refractive index within cells and clustering of AuNVs within endosomes or the cytosol.

Nanocarrier toxicity has been a significant limitation for traditional formulations, such as liposomal or polymeric nanocarriers. To evaluate whether the AuNVs induced cytotoxicity in the DCs, we conducted alamarBlue viability assays using a murine bone marrow-derived dendritic cell line (JAWS II) after incubation with OVA or gp100 AuNVs at various concentrations for 24 h. The fluorescence intensities indicate cellular health and were normalized to the cell control (media only). The viability did not decrease following the addition of AuNVs (ranging from 127% to 155%) when compared to the media-only control (100%) (Additional file 1: Figure S2). Interestingly, the fluorescence intensities for all of the particle-treated JAWS II conditions were significantly higher than the media-only controls (*p* < 0.0015). alamarBlue measures cellular health by cleavage of the metabolite into fluorescent molecules. Improved metabolic activity may increase the amount of fluorescent by-product. Hence, the results suggest that AuNVs may have caused dendritic cell activation by increasing cellular activity, which can also enhance anti-tumor immune responses.

### Functional evaluation and optimization of AuNVs using IFN-γ ELISPOTs

The functional characterization of the AuNVs was performed in two parts: (1) simple splenocyte IFN-γ ELISPOT assays for initial testing and (2) DC-to-splenocyte ELISPOTs for design optimization. Design optimization consisted of four sections: (1) conjugation method optimization, (2) linker optimization, (3) AuNP core size effects, and (4) peptide pool modifications. The ELISPOT assays indirectly measures antigen-specific CD8^+^ CTL ability to secrete IFN-γ, which highly correlates to anti-tumor immunogenicity [6,24]. Gp100 AuNVs were used to stimulate gp100-specific T cells from pmel-1 transgenic mice, while OVA AuNVs were used to stimulate transgenic OT-I mice T cells [25].

**Figure 4:**
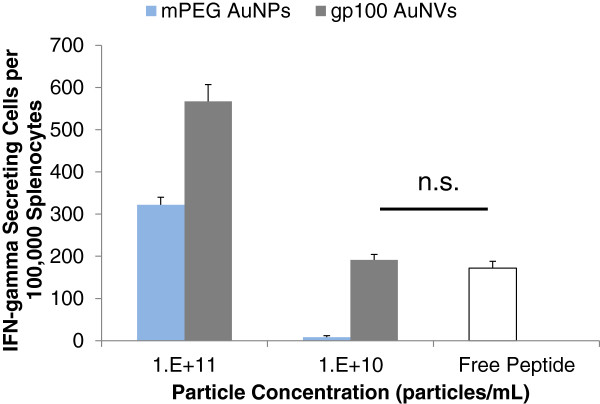
**IFN-γ ELISPOT results from gp100 AuNV induction of pmel-1 splenocytes.** At 10^11^ particles/ml or 25 μg/ml, AuNVs stimulated threefold more IFN-γ secreting cells compared to the free-peptide control. At 10^10^ particles/ml or 2.5 μg/ml maximum dose, the gp100 AuNVs exhibited similar effects as the free-peptide control (10 μg/ml) with no significant difference (*p* = 0.4).

At high particle concentrations (10^11^ particles/ml), gp100 AuNVs were more potent in stimulating pmel-1 splenocytes (567 IFN-γ spot-forming cells (SFC)) compared to mPEG-coated control AuNPs (322 SFC; *p* = 0.005), showing that the linked peptides conjugated on the AuNVs remained functional (Figure 4). At particle concentrations of 10^10^/ml, the AuNVs still had 191 SFC, while the control AuNPs dropped to only 8 SFC. As the particle concentration decreases, the AuNVs still showed an effect up to 10^9^ particles/ml, while at 10^8^ particles/ml, the effects were non-significant relative to the negative controls (media only) (Additional file 1: Figure S3). The AuNV responses were consistently significantly higher (*p* < 0.05) than the responses of the PEG-AuNPs, thus showing that the AuNV effects were not solely caused by the PEG or the AuNPs but due to the peptides conjugated onto the particles (Figure 4). At higher particle concentrations, CTLs may be overloaded with particles, which in turn caused the elevated IFN-γ levels for PEG-AuNP control groups.

For comparative analysis of the efficacy of AuNVs to free peptides, the maximum dose was calculated by multiplying the amount of peptide used to synthesize each particle to the number of particles used. The maximum dose calculation allows a practical evaluation of the cost and benefit of the AuNV design. It would not be overall beneficial if the design required more raw materials than the improvement of the efficacy compared to free peptides. For 10^10^ particles/ml, the maximum dose is calculated to be 2.5 μg/ml. At this particle concentration, the gp100 AuNVs (191 SFC) exhibit similar effects as the free-peptide control (172 SFC) (10 μg/ml) with no significant difference. From this study, we concluded that the AuNVs were able to induce strong IFN-γ release from pmel-1 T cells at approximately fourfold efficiency of the free peptides.

### Optimization of AuNV designs with DC-to-splenocyte IFN-γ ELISPOTs

*In vivo*, antigens (or AuNVs) are uptaken by professional APCs (i.e., DCs), and the TAA-derived peptides are subsequently loaded onto major histocompatibility complexes (MHCs) for stimulation of CD8^+^ CTLs and CD4^+^ helper T cells [26]. Previous ELISPOT assays exposed AuNVs directly to splenocytes, which was a rudimentary way to evaluate the effects of AuNVs. Although there are some antigen-presenting cells in the splenocyte mixture, the result would be occasionally inconclusive.

Thus, to mimic physiological conditions, the AuNVs were incubated with dendritic cells prior to exposure to the splenocytes, eliminating any AuNV direct influence on the splenocytes. The BMDCs were cultured with AuNVs for 24 h. Then, they were washed to remove excess AuNVs and were used as stimulator cells for antigen-specific splenocytes on IFN-γ ELISPOT plates. The DC-to-splenocyte ELISPOT assay can then be used to determine whether the peptides conjugated onto AuNPs can be free for MHC loading.

**Figure 5:**
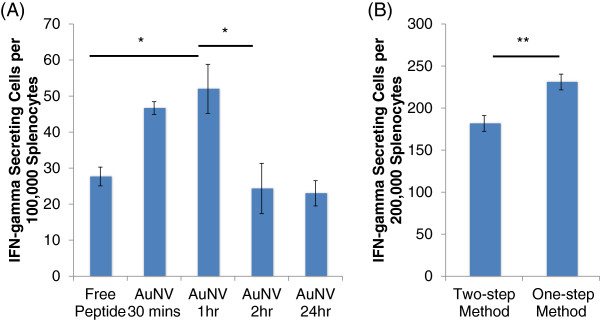
**gp100 AuNVs ELISPOT results for conjugation time optimization and comparison of the two-step and one-step methods.** (**A**) The DC-to-pmel-1 splenocyte ELISPOT results for the gp100 AuNVs at different conjugation times. The 1-h method AuNVs gave the most optimal stimulation results between the various incubation times (single asterisk denotes *p* < 0.05). (**B**) The DC-to-pmel-1 splenocyte ELISPOT results for a comparison of the two-step and one-step method AuNV (double asterisk denotes *p* < 0.01).

Using this model, we evaluated two important factors for improved peptide conjugation onto AuNVs: conjugation duration and scheme. The optimization of conjugation duration is critical for sufficient peptide polymerization while minimizing unwanted cross-linking between the peptide side chains. For conjugation efficiency, we compared the efficacy of AuNVs with varying durations from 30 min to 24 h. Figure 5A shows that AuNVs with 1-h conjugation duration provided the highest IFN-γ secretion (52 SFC). The AuNVs cross-linked for 2 h (24 SFC) were significantly lower than the 1-h particles, while the 30-min AuNVs (47 SFC) were not significantly different from the 1-h AuNVs.

To compare the hydrodynamic particle size of the particles, the DLS data showed that the 1-h conjugation time formed the largest peptide-conjugated AuNVs (approximately 70 nm), which were still much smaller than most liposomal and polymeric formulations (Additional file 1: Figure S4) [8,9]. This advantage can potentially improve lymphatic drainage of the AuNVs. The 2-h AuNVs showed a smaller particle size that supports the hypothesis that synthesis time can cause excessive cross-linkage from the side groups on the peptides and fold on top of the particle.

The scheme used for EDC/sulfo-NHS conjugation is another important factor. As previously mentioned, the conventional two-step conjugation method was designed to minimize affecting the second protein's carboxyls. However, in our situation, enhanced activation of peptide carboxyl groups will be useful for allowing the peptides to link together. Therefore, we designed a new one-step conjugation scheme. The one-step method used lower amounts of EDC/NHS, and the excess linkers were not washed away. In this scenario, the process remained in the MES buffer at pH 6. We hypothesized that the one-step method would be more effective at forming chained peptides, and experimental results support that claim. Using the DC-to-splenocyte IFN-γ assay, the one-step MES buffer method (231 SFC) was significantly more effective in inducing IFN-γ secretion than the two-step design (182 SFC; *p* = 0.004) (Figure 5B). Therefore, the following experiments used the one-step conjugation scheme.

### Comparing efficacies of DNA linker and PEG linker AuNVs

Since PEG is non-degradable, using PEG as linkers raises concern of whether the peptides were released from the AuNPs, which is critical for MHC class I and II loading. To address how peptides are released from the AuNVs following conjugation with the PEG linkers, we examined the degradation of FITC-labeled AuNVs by cell proteases from the JAWS II lysate, an immortalized BMDC cell line. The FITC-labeled AuNVs were incubated with the cell lysate for 24 h. Then, the mixture was centrifuged to remove all the particles and large cell debris. The peptides that came off the particle would then be in the supernatant. Both the OVA and gp100 AuNV showed a twofold increase in the FITC fluorescent levels in the supernatant post-lysate exposure, supporting the hypothesis that cell proteases contained in DCs could remove antigenic peptides from the surface of AuNPs (Additional file 1: Figure S5).

By replacing the non-degradable PEG linkers with poly-T DNA spacers, DNases in the cells would be expected to degrade the spacer and release the peptides from the particle surface. This release mechanism should improve the immunogenicity of the AuNVs. The DNA spacer (11 nt) had a thiol modification on the 5^′^ end and a primary amine on the 3^′^ end. The thiol forms dative bonds with AuNPs in a manner similar to that of PEG-SH. DNA spacers presented amines on the AuNP surface instead of carboxyl groups. Thus, the carboxyl activation step was removed because there were no longer any carboxyl groups on the particles. This change, however, did decrease the peptide conjugation yield.

**Figure 6:**
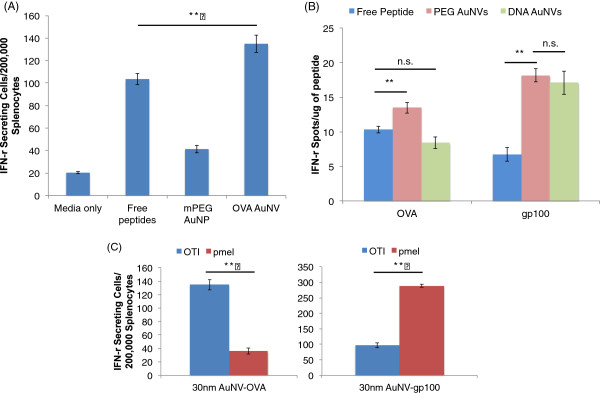
**ELISPOT results comparing efficacies of AuNVs using the modified DC-to-splenocyte assays.** (**A**) The DC-to-OT-I splenocyte IFN-γ ELISPOT data comparing the efficacies of the OVA AuNVs and the free peptides. The AuNVs (max dose 10 μg/ml) were able to induce a significantly stronger response than the free peptides (10 μg/ml) (double asterisk denotes *p* < 0.01). (**B**) The DC-to-OT-I and pmel-1 splenocyte IFN-γ ELISPOT data comparing the PEG linker AuNVs and the DNA spacer AuNVs for OVA and gp100 (double asterisk denotes *p* < 0.01; n.s, not significant). (**C**) The DC-to-OT-I and pmel-1 splenocyte IFN-γ ELISPOT with the OVA and gp100 AuNVs. Each particle responded to its corresponding splenocyte significantly more than the unmatched AuNV (double asterisk denotes *p* < 0.01).

As previously mentioned, the BMDCs were incubated with the AuNVs to minimize non-specific IFN-γ secretion, Then, the loaded BMDCs were exposed to antigen-specific splenocytes, i.e., OT-I for OVA peptides and pmel-1 for gp100 peptides. When the AuNV-loaded DCs were exposed to OT-I splenocytes, the AuNVs (134 SFC), maximum dose of 10 μg/ml, showed significant improvement in IFN-γ secretion compared to the free-peptide control (10 μg/ml; 103 SFC) (Figure 6A). Although the improvement of the OVA AuNVs compared to the OVA free peptides was not as large relative to the gp100 AuNV samples, both tested AuNVs showed a significant improvement in the splenocyte response compared to the free peptides (*p* < 0.01) (Figure 6B).

To visualize the effects of AuNVs, we standardized the ELISPOT spot count with the amount of peptide used. The ELISPOT results in Figure 6B show that the DNA spacers (8 SFC/μg) do not work as well as the PEG linker (14 SFC/μg) for OVA on the AuNVs because their effects were similar to those of free peptides (10 SFC/μg). However, the DNA spacer (17 SFC/μg) and PEG linker AuNVs (19 SFC/μg) for gp100 showed significant improvement in CTL stimulation.

To verify that the splenocyte IFN-γ induction is specific to the correct peptides, we exposed the OVA and gp100 AuNVs to BMDCs and then exposed them to OT-I splenocytes and pmel-1 splenocytes (Figure 6C). From the ELISPOT results, the OT-I splenocytes responded significantly more to the OVA AuNVs (135 SFC) than the gp100 AuNVs (96 SFC) and vice versa for pmel-1 (OVA, 36 SFC; gp100, 289 SFC). Therefore, in addition to being simple, versatile, and cost-effective, our AuNV design is highly specific and non-toxic.

### AuNV evaluation with various particle core sizes

As noted above, the hydrodynamic particle size of the AuNV can be important for particle migration to lymph nodes. The AuNV size can be controlled by the size of the core AuNP. We used DC-to-OT-I splenocyte ELISPOTs to measure the size effects from 15-, 30-, and 80-nm OVA AuNVs. This assay cannot test the subcutaneous draining abilities of the AuNV particles and would require an *in vivo* study to select the best core size. However, the results suggest that particle size does not significantly alter the IFN-γ efficacy using *in vitro* assays (Additional file 1: Figure S6). All three particle size conditions had a maximum peptide dose of 10 μg/ml, which correlates to 7 × 10^11^ particles/ml for 15-nm cores, 10^11^ particles/ml for 30-nm cores, and 5.5 × 10^9^ for 80-nm cores.

### Peptide-pool AuNVs

To this point, the AuNV designs have been focused on using MHC class I peptides. Although most vaccine work has been focused on specific CD8^+^ T cells epitopes, individual epitopes for all HLA types for MHC I and II have not been identified. However, peptide pools are segments with overlapping amino acid (aa) sequences that incorporate an entire antigen sequence.

The versatility of the AuNV design allows for conjugation of longer peptides or mixtures of peptides (i.e., peptides pools) from different tumor antigens onto AuNPs. The gp100 peptide pool, for example, has peptides that are 15 aa in length with 11 aa overlaps. Including the entire antigen sequence has three extra advantages: (1) the natural cleavage sites are present to facilitate peptide release from the particles, (2) both the MHC class I and II epitopes are included, and (3) peptide pools are easily synthesized and can replace expensive and time-consuming recombinant whole-protein isolation.

The gp100 peptide pool AuNVs were used in the DC-to-pmel-1 splenocyte ELISPOTs, and the results show that the average number of spots for the peptide pool AuNVs was higher than that for the free-peptide pool (Additional file 1: Figure S7). However, the peptide pool AuNVs exhibited a much larger standard error and had a non-significant difference between the AuNVs and the free peptides (*p* = 0.34). This is because the assay only evaluated one specific MHC class I epitope by using pmel-1 splenocytes. Peptide-pool AuNVs may have several other benefits that were not tested here, such as helper T cell responses and facilitating peptide separation from the particles due to preserved natural cleavage sites. These effects may be very useful in *in vivo* settings.

## Discussion

Gold nanoparticles are unique nanomaterials that are easy to synthesize and modify. AuNPs have excellent optical properties that can be exploited for detection or photothermal applications. In addition, AuNPs accumulate in phagocytic cells such as macrophages and dendritic cells, making them ideal vehicles for vaccine delivery. Here, we demonstrated a method to synthesize high-peptide density gold nanovaccines using a simple self-assembling bottom-up strategy. Changes in the absorbance spectra and TEM images show successful peptide conjugation onto PEGylated AuNPs. Calculating from the conjugation yield of 90%, each particle can carry up to 1,300 peptides. Moon et al. [27] reported liposomal formulations to have an encapsulation efficiency of 200 to 350 μg OVA/mg of particles and poly(lactic-*co*-glycolic acid) formulations to have 50 μg OVA/mg of particles, while AuNVs correlate to roughly 500 μg of OVA peptide per milligram of AuNVs. Considering that gold also has a higher density than liposomal or polymeric formulations, the amount of peptide carried by AuNVs is much higher than that by other nanomaterials.

Not only does AuNVs have high peptide density, but we also observed that AuNV behavior in solution depends on the properties of the peptides that were used for conjugation. The OT-I peptides from the antigen OVA are neutral in charge with an isoelectric point near physiological pH (6.34). Thus, OVA AuNVs were easily suspended in PBS. Ninety-four percent of the OVA AuNVs were recovered throughout the multiple centrifugation and washing steps with PBS. In comparison, the Trp-2 peptides are 78% hydrophobic. The Trp-2 AuNVs adhered to the hydrophobic polypropylene tube after peptide conjugation was completed and did not detach during centrifugation or probe sonication. However, the Trp-2 AuNVs remained in solution when ethanol (0.2% *v*/*v* Tween 20) was added to the tubes due to the decrease in polarity of the solvent and the addition of surfactants (Additional file 1: Figure S8). Thus, AuNV particle behavior in solution is dependent on the peptide properties.

Having high peptide density on AuNVs is important for vaccine function because the peptide-coated nanocarriers collect in the endosomes and can mimic the size of pathogens, stimulating DC maturation. The induction of DCs to mature and to present tumor antigens is crucial for engineering a successful vaccine. This stimulation by nanomaterials has been shown by Moon et al. to cause DCs to induce large amounts of cross-presentation for stronger and sustained anti-tumor immune responses [27]. Cross-presentation is very important for CTL stimulation because it is required to allow peptides to enter the MHC class I (cytosolic) pathway from the MHC class II (endosomal) pathway. By using MHC class I peptides, DC-to-splenocyte ELISPOTs can be used to evaluate the extent of cross-presentation. Additionally, the assay itself is of interest because it can screen large numbers of nanovaccines *in vitro*, simulating the process of antigen presentation and preventing extensive use of animals.

Once the AuNVs enter the endosomes, it is critical that the peptides can come off the particles and enter the MHC class I pathway. Therefore, the conjugation optimization of conjugation duration and schemes is a key for an effective AuNV. From the optimization results, we concluded that the 1-h conjugation time was most effective. We hypothesize that the peptides link linearly during the 1-h conjugation but will begin to cross-link transversely via peptide side groups by 2 h. The non-linear cross-linking could disrupt the peptide sequence or presentability, thus lowering the efficacy and size of those AuNVs.

As for the method optimization, the buffers used for the conjugation process cause a significant impact on the AuNV efficacy. MES buffer has a pK_a_ of 6.15, which is within the range for EDC coupling to carboxyl groups generating *O*-acylisourea [28,29]. Sulfo-NHS was then added to replace the *O*-acylisourea to form semi-stable amine-reactive NHS esters. Amine binding to the NHS esters reacts better at neutral to higher pH [29]. Thus, switching to PBS at pH 7.4 prevents excessive self cross-linkage. Furthermore, the one-step (MES) method allows better carboxyl activation and a higher chance of extra linkages or cross-linkage, but it can also cause excessive cross-linkages from the side changes of the peptides, which can lower the efficacy of the vaccine peptides. Conversely, the two-step method (MES-PBS) allows less side chain linkage but lowers overall peptide linkage. From the results, the one-step method AuNVs were significantly better at stimulating CTLs than the two-step method. Additionally, the one-step method is more time- and cost-efficient because it requires fewer reagents and less processing for improved overall yield of particle collection.

After optimizing the conjugation time and method, we investigated effects of different linkers such as DNA, which should be degraded intracellularly and allow peptide layers to be released from the gold surface. However, the results show that PEG linker-based AuNVs were significantly more effective at stimulating CTLs. The decrease in efficacy for the DNA-linked OVA AuNVs is probably due to two factors. First, the lack of activated carboxyl groups (i.e., PEG linker AuNVs) results in the deficiency to form polymerization points. Therefore, insufficient peptide polymerization is caused by excessive peptide self-polymerization off the AuNPs to form small peptide clumps in the solution. Second, there is a reduced amount of linkers on the AuNPs because the DNA spacer requires more foot space than the PEG linker [30,31]. Overall, the data here suggest that the PEG linker design provides the best AuNVs for both peptide types.

## Conclusions

In conclusion, improving vaccine delivery using nanocarriers can stimulate a sustained anti-tumor response while inducing activation and maturation of DCs. Here, we designed AuNVs by self-assembling modified PEGs and tumor-associated antigen peptides on gold nanoparticle surfaces. AuNVs carry large doses of peptides by using a simple bottom-up conjugation strategy to layer peptides onto the PEG-modified AuNPs. We showed that the simple AuNV design improved *in vitro* immune cell stimulation while maintaining a sub-100-nm diameter size to allow effective delivery and improve immunogenicity of vaccine antigen peptides such as ovalbumin and gp100.

## Abbreviations

APC: Antigen-presenting cell;AuNP: Gold nanoparticle;AuNV: Gold nanovaccine;BMDC: Bone marrow-derived dendritic cell;CTL: Cytotoxic lymphocytes;DC: Dendritic cell;DLS: Dynamic light scattering;ELISPOT: Enzyme-linked immunosorbent spot;IFN: Interferon;i.v.: Intravenous;MHC: Major histocompatibility complex;PEG: Polyethylene glycol;s.c.: Subcutaneous;SFC: Spot-forming cell;TAA: Tumor-associated antigens;TEM: Transmission electron microscopy

## Competing interests

The authors declare that they have no competing interests.

## Authors’ contributions

AL developed the project including the particle design and conducted the *in vitro* cellular experiments. He conducted the statistical analysis and wrote the manuscript. JL, AB, and PE assisted in the development of the experiments. JY provided consultation for the nanoparticle conjugation and physics. LL assisted in the particle synthesis. AF and RD guided the project and oversaw the manuscript preparation. All authors read and approved the final manuscript.

## Supplementary Material

Additional file 1 **Supplementary information.** Description: A document containing eight supplementary figures and one supplementary table. Click here for file
