# Supplementary material for: High-density sub-100-nm peptide-gold nanoparticle complexes improve vaccine presentation by dendritic cells in vitro
Source: Nanoscale Res Lett. 2013 Feb 12;8(1):72. doi: 10.1186/1556-276X-8-72 (PMC3579702; doi:10.1186/1556-276X-8-72)
Supplement: Additional file 1 — Supplementary information. Description: A document containing eight supplementary figures and one supplementary table. [file 1556-276X-8-72-S1.docx]

**Supplementary Information**


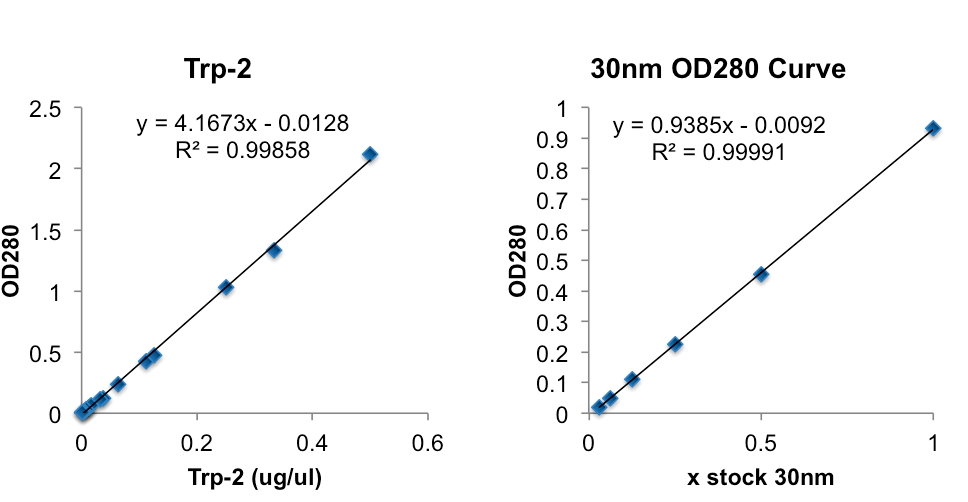


**Figure S1**. Absorbance 280nm standard curves, free Trp-2 peptides (left) and gold nanoparticles (right), were used to calculate conjugation yield.

**Figure S2.** The alamarBlue fluorescence (viability) reading of JAWS II cells after a 24-hour incubation with AuNVs in comparison to the media only control. The particles added to the solutions were at 10^11^ particles/ml. All particle conditions were significantly higher than the media control (p<0.0015).


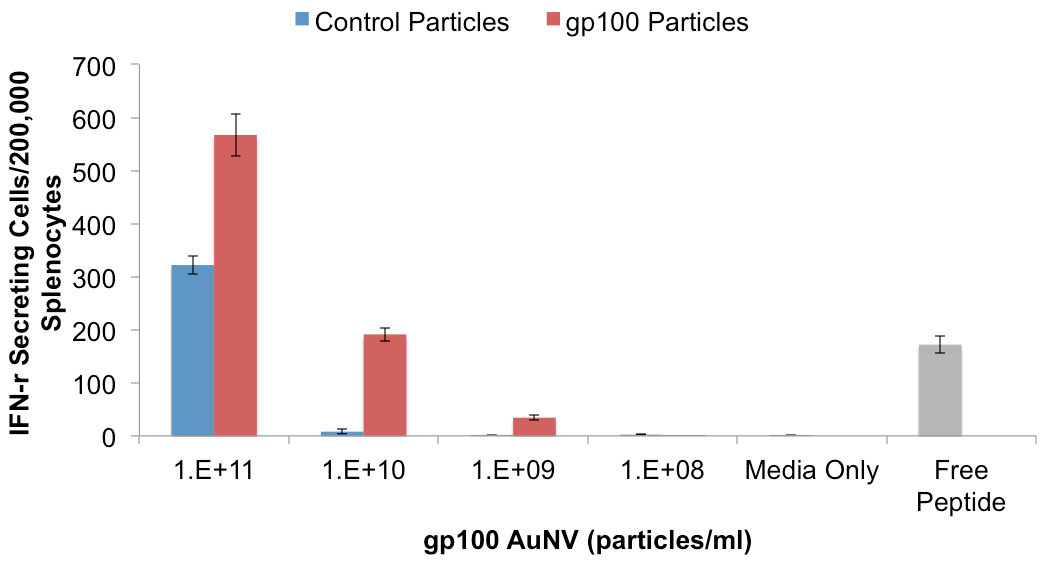


**Figure S3.** IFN-γ release ELISPOT results from gp100 AuNV induction with Pmel-1 splenocytes. Particle concentrations were in particles/ml. Media only condition served as a negative control while free peptides condition used 10μg gp100 peptides/ml.


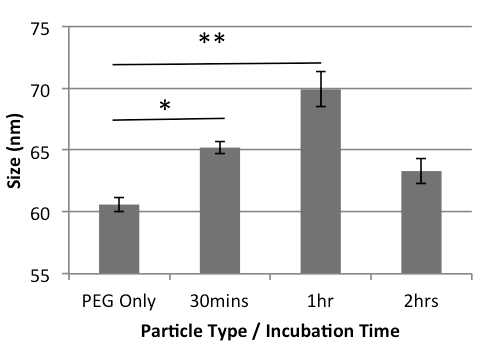


**Figure S4.** Dynamic light scattering (DLS) size reading of AuNPs only functionalized with PEG-COOH or additionally functionalized with peptide through EDC/NHS chemistry for various crosslinking times. Time points (30mins, 1 hr, and 2 hrs) refer to crosslinking times of the peptide and EDC/NHS activated particles before addition of hydroxylamine to quench the reaction. Both 30mins (*) and 1hr (**) incubation time particles have a significantly different size than the preconjugate 30nm-PEG-COOH particles. *: p<0.05, **: p<0.01


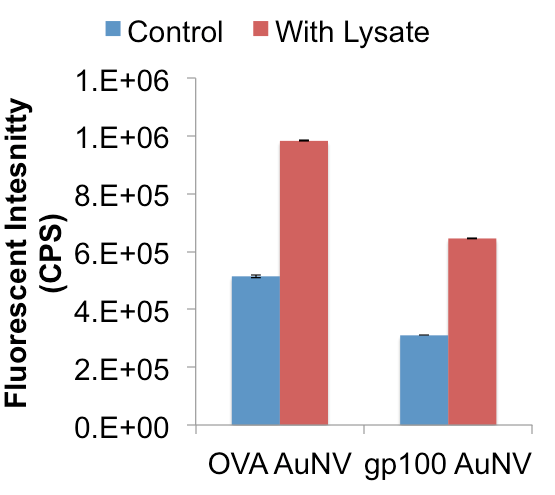


**Figure S5.** Fluorescence intensities of fluorescent OVA and gp100 AuNVs with or without JAWS II cell lysate incubation for 24 hrs. The lysates increased the fluorescence intensity by two-fold for the OVA and gp100 AuNVs.


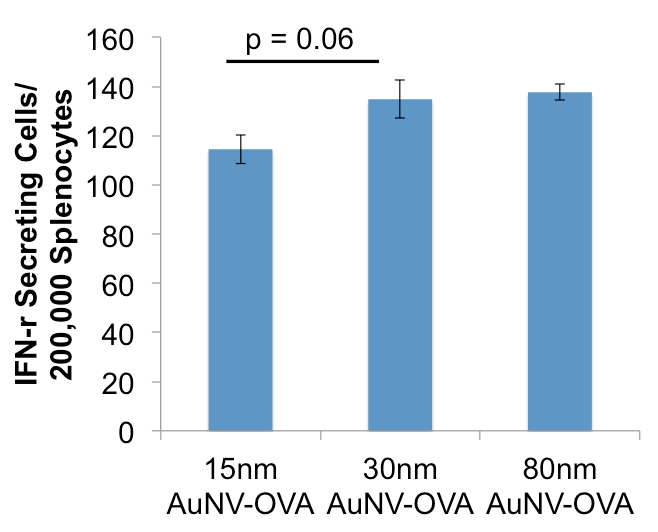


**Figure S6.** The DC-to-OT-I splenocyte IFN-γ ELISPOT results using 15-nm, 30-nm and 80-nm AuNPs as the starting cores. All three showed similar results. The number of IFN-γ secreting cells per 200,000 splenocytes for 15-nm AuNVs was not significantly lower than that for the 30-nm AuNVs.


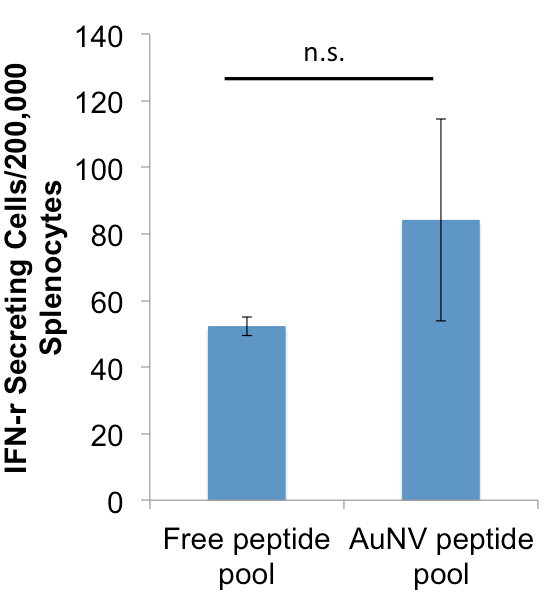


**Figure S7.** The DC-to-pmel-1 splenocyte IFN-γ ELISPOT using free and AuNV gp100 peptide pools. The peptide pool AuNVs showed a very large standard error of mean (SEM), being ten-fold greater than the free-peptide pool’s SEM.


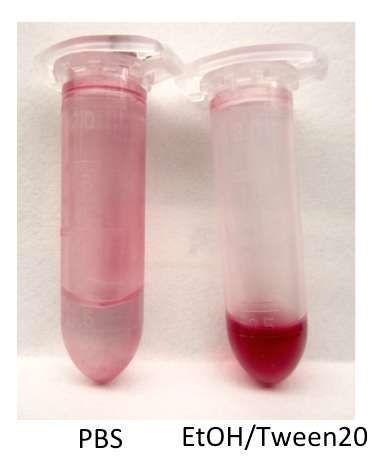


**Figure S8.** Trp-2 AuNVs in PBS (left) and ethanol (right). Trp-2 peptides are very hydrophobic (78%). Trp-2 AuNVs do not dissolve in PBS and thus stick to the sides of the tube. However, Trp-2 AuNVs do resuspend in ethanol with 0.2% Tween 20.

**Table S1.** Properties of vaccine peptides used. (Genescript Peptide Property Calculator) https://www.genscript.com/ssl-bin/site2/peptide_calculation.cgi

| Peptide | Sequence | Charge | Isoelectric Point | Components |
| --- | --- | --- | --- | --- |
| OVA | SIINFEKL | 0 | 6.34 | Hydrophilic: 25%  Hydrophobic: 50%  Others: 25% |
| gp100 | KVPRNQDWL | 1 | 9.71 | Hydrophilic: 33%  Hydrophobic: 33%  Others: 33% |
| Trp-2 | SVYDFFVWL | -1 | 3.75 | Hydrophilic: 11%  Hydrophobic: 78%  Others: 11% |
